# Time-Resolved Pump-Probe X-Ray Solution Scattering Capabilities at BioCARS 14 ID Beamline, Advanced Photon Source.

**DOI:** 10.1063/4.0000925

**Published:** 2025-10-27

**Authors:** Irina Kosheleva, Robert Henning, Insik Kim, Eric Zoellner, Vukica Srajer, Rama Ranganathan

**Affiliations:** The University of Chicago, Chicago, IL, USA

## Abstract

A fundamental problem in biological sciences is to connect biological function of a molecule with its structure. This is typically achieved by initiating a reaction in a biomolecule, and then following structural changes. Here we present time-resolved capabilities in X-ray solution scattering at BioCARS, National User Facility and a synchrotron resource for structural dynamic in biology, located at 14-ID beamline at Advanced Photon Source. At BioCARS, solution scattering experiments are conducted with the polychromatic X-ray beam of 12 keV , 2.5% - 4.5% bandpass, beam size at the sample position of 15x25 (VxH) μm^2^, and exposure times from 250 ps up to 7 μs (from 5.8 *10^9^ photons per single 250 ps X-ray pulse to 5.5 *10^11^ photons per 7 μs pulse train). BioCARS solution scattering setup is suitable for experiments with Q-range of ∼0.01-5 1/Å and allows studies of molecules up to 300 Å in size. At BioCARS, we use high-power pulsed UV, VIS and IR lasers to initiate a reaction. The reaction may be initiated in a number of ways: by absorbing a laser pulse by a chromophore in a photosensitive solute molecule, by using heating of the solvent by an IR laser pulse, or by using a laser pulse to de-cage small functional molecules which then initiate the reaction. By using laser pulses for reaction initiation, we can obtain structural information of biomolecule undergoing the reaction with time-resolution of 250 ps and longer. Typical examples of time-resolved solution scattering experiments at BioCARS are studies of proteins following their basic functions such as folding, binding, signal transduction, and allosteric motion.

